# 2015 ISCB Overton Prize Awarded to Curtis Huttenhower

**DOI:** 10.1371/journal.pcbi.1004319

**Published:** 2015-06-11

**Authors:** Christiana N. Fogg, Diane E. Kovats

**Affiliations:** 1 Freelance Science Writer, Kensington, Maryland, United States of America; 2 International Society for Computational Biology, La Jolla, California, United States of America

The International Society for Computational Biology (ISCB) honors the achievements of an early- to mid-career scientist with the Overton Prize each year. The Overton Prize was instituted to honor the untimely loss of Dr. G. Christian Overton, a respected computational biologist and founding ISCB Board member. Winners of the Overton Prize are independent investigators in the early to middle phases of their careers who are selected because of their significant contributions to computational biology through research, teaching, and service.

ISCB is pleased to recognize Dr. Curtis Huttenhower, associate professor of computational biology and bioinformatics at the Harvard T. H. Chan School of Public Health, as the 2015 winner of the Overton Prize. Huttenhower will be presenting a keynote presentation at the 2015 Annual International Conference on Intelligent Systems for Molecular Biology/European Conference on Computational Biology (ISMB/ECCB) in Dublin, Ireland, in July 2015.

## Curtis Huttenhower: From Linguistics to the Gut

**Fig 1:**
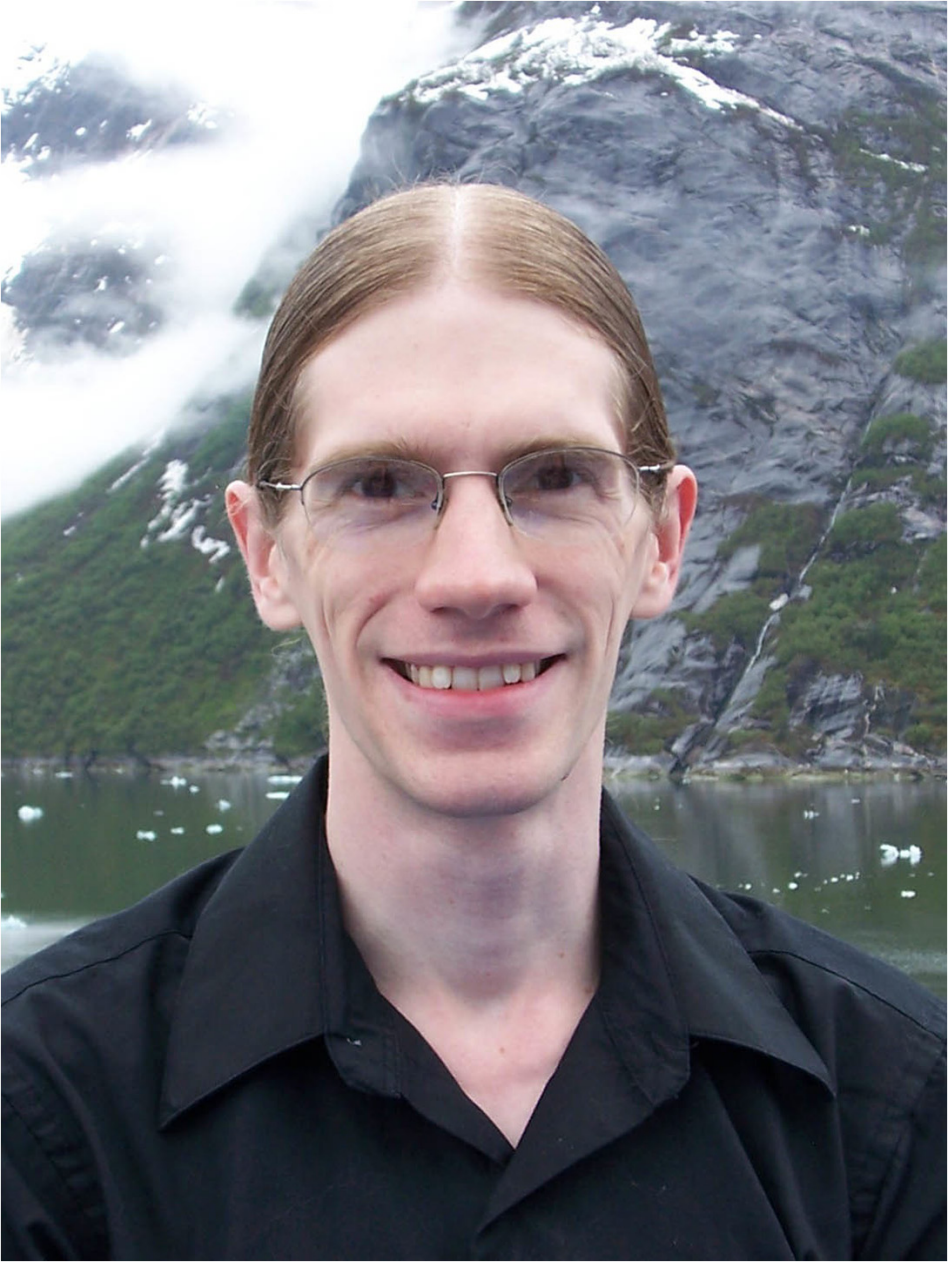
Curtis Huttenhower. Image credit: Broad Institute of Massachusetts Institute of Technology (MIT) and Harvard, Cambridge, Massachusetts, United States of America.

Curtis Huttenhower (Fig 1) has vivid memories of his Apple IIe, the tool that first brought him into the world of computer science by way of Beginner's All-purpose Symbolic Instruction Code (BASIC) programs and text adventure games like Zork. His first research experience was in computational linguistics, and he recalls fondly how the computer games he played as a child were text driven and quite different than modern games' graphical experiences. Huttenhower’s early experiences "talking" with computers drew him to later study computational language processing, and he recalled, “Some of the same tricks those games used to communicate in the ‘80s are still in use today. It's surprisingly challenging for a computer to understand sentences like "Pick up the red fruit" in reference to an apple, when a human child has no problem.”

Huttenhower graduated in 2000 from the Rose-Hulman Institute of Technology, where he majored in computer science, chemistry, and math. He remembers being drawn to the physical sciences as well as computation, but he also admitted that despite his interest in the natural sciences, he was at first discouraged from studying these fields because he was dreadful at memorization. He recounted, “I was, and remain, absolutely terrible at memorizing lists of facts. That kept me out of biology for a long time. It's a rare student who enjoys the toughest parts of general biology or chemistry, memorizing gene and chemical names. Organic chemistry was all about rules rather than facts, though—generalizable principles. That got me into biochemistry, and the combination of problem solving in the wet lab and problem solving in the dry lab, by way of bioinformatics, was a lot of fun.”

Although Huttenhower graduated with excellent undergraduate academic performance, he was initially rejected from all the graduate programs he applied to, and he found himself going in a very different direction by taking a software development job with Microsoft. In retrospect, though, he considers his two years at Microsoft as an invaluable experience, during which he learned the importance of management and testing infrastructure and the value of standard operating procedures and standardized methods for organizing computational projects. Huttenhower also sees value in this experience as he advises trainees about career options in academia and industry. "Particularly given the challenges of the modern life science careers," he mentions, "it's important to remember how productive and enjoyable industry work can be, and that neither of the two options are intrinsically better or worse than the other."

Huttenhower was finally accepted into a graduate program in computational linguistics at Carnegie Mellon University after two years of applications, and he has a new perspective on this process now. “Serving on admissions committees a decade later,” he said, “I understand better now what a challenging process it is.” Dannie Durand was Huttenhower’s research advisor, and he credits her for his current career path. He recalled, “She was a joy to work with and tremendously enthusiastic about bioinformatics, and the enthusiasm was catching. By the time I finished my MS, I was applying for PhD programs in computational biology rather than language processing. She and Russell Schwartz were, and still are, fantastic motivators of new students entering the field.”

Huttenhower then went to Princeton University to pursue his PhD and postdoctoral studies in computer science under the guidance of Olga Troyanskaya. He credited Troyanskaya’s mentorship as elemental to his success, and he described, “[She] created, and has since grown, an exciting and productive lab. In addition to key pieces of basic knowledge in bioinformatics and strategies for a research career, I learned from her how to figure out which scientific problems matter, at least inasmuch as any of us know.”

In 2009, Huttenhower accepted a position as an assistant professor of computational biology and bioinformatics in the Department of Biostatistics at the Harvard T. H. Chan School of Public Health. He considers himself fortunate to have great mentors, especially Owen White, John Quackenbush, and Ramnik Xavier, as he has launched his academic career. Huttenhower has also considered his friendships and collaborations with other junior faculty as crucial to early survival. He sees now that this mutual support and guidance has been pivotal in the success of early career computational biologists. “Chad Myers, Matt Hibbs, Florian Markowetz, and Edo Airoldi formed the core of Olga's lab along with me in her first few years as faculty,” he recalled, “and they've all gone on to extremely successful research careers."

Huttenhower has established a robust research program in his lab, with a large part of his group working on projects related to the National Institutes of Health (NIH) Human Microbiome Project. He considers microbiome research to be an area of particular interest because relatively little is known about microbial communities, the field has a broad and solid foundation in classical microbiology on which to build, and findings in this area may significantly influence human health. He is enthusiastic about translating microbiome research to clinical applications. He said, “The microbiome represents an untapped new source of possible disease biomarkers and therapeutic interventions. In inflammatory bowel disease and type 1 diabetes, we're trying to determine which changes in the microbiome predict disease onset or inflammatory activity. Even in the worst case, knowing when a flare or seroconversion was becoming likely would allow stronger treatments to be introduced to prevent exacerbation or stave off disease activity. In the best case, the microbiome is modifiable, unlike human genetics. It's difficult to modify, since like cancer it represents a complex system that's evolved to be resistant to change, but the potential is there.”

Huttenhower is excited about his work, and he shares a good problem to have with other life sciences researchers. “Like many scientists in public health,” he said, “we spend our time working both on translational applications and on interesting basic biology, and at this point in the field as a whole there are many findings from both areas that represent ongoing work with a lot of potential.” The tools developed for his lab’s human microbiome research can also be applied to research on other microbial communities, and there are many open questions in their uses for areas as far-flung as agricultural microbial communities, bioremediation, and the basic biology of microbial interactions. Huttenhower noted, “*Saccharomyces cerevisiae* has been a great model microbial isolate for decades, and we're still learning how to make better wine, beer, and bread, so research into the complexities of mixed microbial communities is unlikely to run out of challenges any time soon!”

